# Effects of long-term use of macrolides in patients with non-cystic fibrosis bronchiectasis: a meta-analysis of randomized controlled trials

**DOI:** 10.1186/s12879-015-0872-5

**Published:** 2015-03-27

**Authors:** Li-Chao Fan, Hai-Wen Lu, Ping Wei, Xiao-Bin Ji, Shuo Liang, Jin-Fu Xu

**Affiliations:** Department of Respiratory Medicine, Shanghai Pulmonary Hospital, Tongji University School of Medicine, No. 507 Zhengmin Road, Shanghai, 200433 China

## Abstract

**Background:**

The purpose of this study was to evaluate the clinical benefits and safety of the long-term use of macrolides in patients with non-cystic fibrosis (non-CF) bronchiectasis.

**Methods:**

Embase, Pubmed, the Cochrane Library and Web of Science databases were searched from inception up to March 2014. The primary outcome was the improvement of exacerbations of bronchiectasis. Secondary endpoints included changes of microbiology, lung function, quality of life, sputum volume, adverse events and macrolide resistance.

**Results:**

The literature search yielded 139 studies, ten of which containing 601 patients were included in this meta-analysis. Macrolides showed a statistically-significant improvement in reducing acute exacerbations per patient during follow-up treatment (RR = 0.55, 95% CI: 0.47, 0.64, P < 0.001), increasing the number of patients free from exacerbations (OR = 2.81, 95% CI: 1.85, 4.26, P < 0.001), and prolonging time to a first exacerbation (HR = 0.38, 95% CI: 0.28, 0.53, P < 0.001). Macrolides maintenance treatment was superior to control with respect to attenuating FEV1 decline (p = 0.02), improving sputum volume (p = 0.009) and SGRQ total scores (p = 0.02), but showed a higher risk of adverse events, especially diarrhea (OR = 5.36; 95% CI: 2.06, 13.98, P = 0.0006). Eradication of pathogens was improved in the macrolide group (OR = 1.76, 95% CI: 0.91, 3.41, P = 0.09), while pathogen resistance caused by macrolides dramatically increased (OR = 16.83, 95% CI: 7.26, 38.99, P < 0.001). The new appearance of a microbiologic profile or participant withdrawal due to adverse events showed no significant differences between the two groups.

**Conclusion:**

In patients with non-CF bronchiectasis, macrolide maintenance treatment can effectively reduce frequency of exacerbations, attenuate lung function decline, decrease sputum volume, improve quality of life, but may be accompanied with increased adverse events (especially diarrhea) and pathogen resistance.

**Electronic supplementary material:**

The online version of this article (doi:10.1186/s12879-015-0872-5) contains supplementary material, which is available to authorized users.

## Background

Non-cystic fibrosis (non-CF) bronchiectasis is a respiratory disease characterized by persistent airway inflammation and dilation of the bronchial wall driven by various causes [1]. Patients with bronchiectasis suffer from sputum production, recurrent exacerbations, and progressive airway destruction [2]. From 2000 to 2007, the prevalence of bronchiectasis in the United States was 1,106 cases per 100,000 with an annual percentage increase of 8.74% [3]. The average annual hospitalization rate was 9.4 per 100,000 in Germany during 2005–2011, with the highest rate reaching 39.4 hospitalizations per 100,000 among men aged 75–84 years [4].

Major therapy for bronchiectasis is focused on breaking the “vicious cycle” of mucus stasis, infection, inflammation, and airway destruction [5,6]. Accumulating evidence shows that macrolides possess immune-regulatory and anti-inflammatory functions beyond their anti-microbial effects [7-10]. Macrolide antibiotics have been effectively used in the treatment of diffuse panbronchiolitis, COPD and cystic fibrosis [11-14]. It remains uncertain how well macrolides can serve in the management of non-CF bronchiectasis. More recently, the effects of macrolide antibiotics have been reported to be mainly positive in non-CF bronchiectasis albeit with variable results. However, there remain many unanswered questions due to small sample size and study design. This prompted us to systematically assess the effects of these drugs on patients with non-CF fibrosis bronchiectasis. The present meta-analysis was undertaken to determine the efficacy and safety of macrolide maintenance therapy in non-CF bronchiectasis patients.

## Methods

This review was registered in PROSPERO (CRD42013004656) (Additional file 1) and performed adhering to PRISMA guidelines (Additional file 2).

### Search strategy

Pubmed, Embase, Web of Science and the Cochrane Library were comprehensively searched from inception to March, 2014 by two investigators (L-CF and J-FX), respectively. No language restriction was applied. A Keyword Search included “Macrolides” or “azithromycin” or “erythromycin” or “clarithromycin” or “roxithromycin” and “bronchiectasis” or “non-cystic fibrosis bronchiectasis” or “non-CF bronchiectasis” or “NCFB” and “randomized controlled trial” or “RCT”. In addition, relevant articles were manually searched and reviewed.

### Study selection

The two reviewers (L-CF and H-WL) independently searched the literature and identified relevant articles for further assessment of data on efficacy and safety. A study was considered eligible if (1) it was a clinical randomized controlled trial (RCT); (2) it assessed the efficacy or safety of macrolides in comparison with placebo, another class of antibiotic or blank control in the treatment of patients with non-CF bronchiectasis. A study was excluded if (1) it presented as a review article or protocol; (2) involved patients with chronic respiratory conditions other than non-CF bronchiectasis, such as cystic fibrosis, COPD, asthma; (3) the duration of treatment was less than 8 weeks; or (4) the data could not be extracted with current mathematical methods.

### Assessment of validity

A quality assessment of each study was performed by SL and X-BJ independently according to the Cochrane Collaboration tool in the Review Manager software. The details of quality review included: (1) random sequence generation (selection bias); (2) allocation concealment (selection bias); (3) blinding of participants and personnel (performance bias); (4) blinding of outcome assessment (detection bias); (5) incomplete outcome data (attrition bias); (6) selective reporting (reporting bias); (7) other bias. Disagreements were resolved by consensus or by a third reviewer.

### Data extraction

Two reviewers (PW and H-WL) independently evaluated all eligible studies and extracted relevant data. From each eligible study, a variety of characteristics were recorded, including study location, design, number of patients (male/female), mean age, intervention and duration. The primary outcome assessed was the changes of non-CF bronchiectasis exacerbations. A bronchiectasis exacerbation was defined as deterioration in cough, dyspnea, wheezing, fever, chest pain, increased purulent sputum, requirement for oral or intravenous additional courses of antibiotics. Secondary outcomes included: quality of life, lung function, sputum volume, the degree of dyspnea, adverse events, participants withdrawn due to side effects, changes of microbiologic profile in sputum or bronchoalveolar lavage fluid (BALF) or nasal swab, and macrolide resistance. Attempts were also made to contact authors for necessary information. If they were not provided, they were calculated using provided study data. Discrepancies were resolved by a consensus.

### Statistical analysis

The meta-analysis were performed with Review Manager software (version 5.2; Cochrane Collaboration, Oxford, United Kingdom) and Stata Statistical software (version 12.0; Stata Corporation, College Station, TX, USA). Most items of the meta-analysis were performed with Review Manager software and OR was used as a measure for dichotomous. Only the outcomes of time to a first exacerbation and the number of acute exacerbations per patient were calculated by Stata Statistical software, for publications did not provide enough data to calculate mean difference (MD) and 95% CIs in Revman. We only obtained the data of hazard ratio (HR) and rate ratio (RR) with 95% CI, respectively. The exact methodology to pooled the data by Stata was according to Le and Parmar, using the command “metan lnHR lnll lnul, eform label (namevar = Study) boxsca (0.9) random xlabel(0.5,1,1.5) effect (“HR”) texts (250) “to pool the studies. Weighted mean difference (WMD) or standard mean difference (SMD) are used for meta-analysis of continuous data. Statistical heterogeneity among studies was determined by Cochran’s *x*^2^ statistics with P value and I^2^ throughout the meta-analysis. The value of p <0.10 or I^2^ > 50% was suggestive of significant heterogeneity, in which case we chose a random-effects model [15]. Otherwise, calculations were performed with a fixed-effects model. If substantial heterogeneity was identified, subgroup analysis was performed to explore heterogeneity. The publication bias was assessed by the funnel plot.

## Results

### Literature search

**Figure 1:**
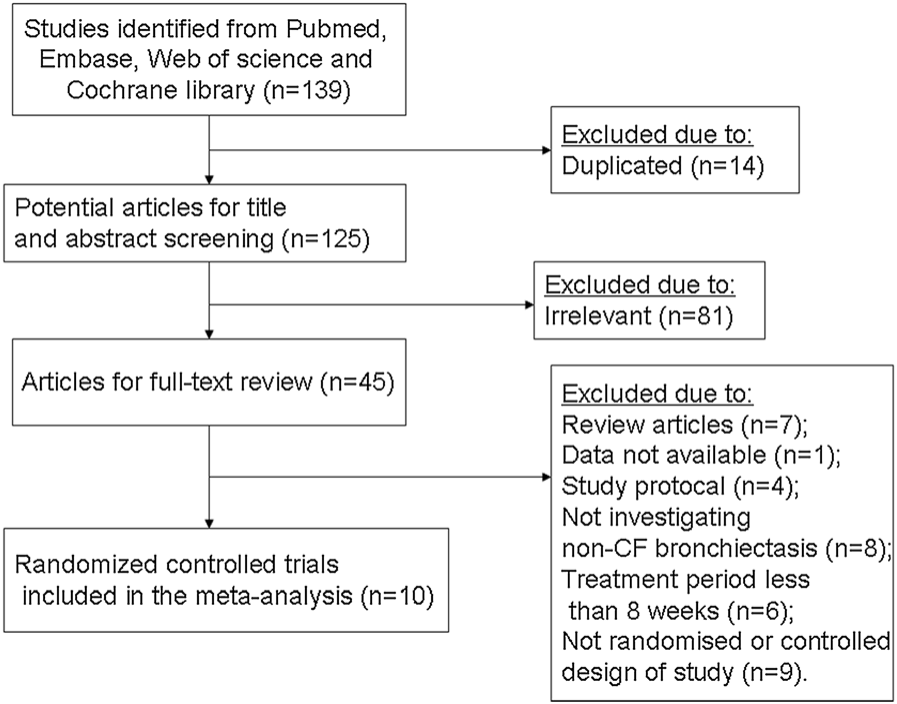
Flow diagram of the process of selection of included studies. Flow chart depicts the selection process at each stage.

Our initial search identified 55 potentially relevant studies in Pubmed, 39 studies in Web of Science, 37 studies in Embase and 8 articles in the Cochrane Library. After screening titles and abstracts, 45 papers were considered potentially eligible for full text review. Of these, 35 were excluded for various reasons, and 10 RCTs [16-25] were identified for meta-analysis (Figure 1).

### Characteristics of included trials

The characteristics of the eligible studies were summarized in Table S1 (see Additional file 3). These studies were conducted from 1995 to 2011. Both children and adult patients were included. Patients were either infected with *P. aeruginosa* or other pathogens. Of all the RCTs included in the meta-analysis, five studies used azithromycin in the treatment group [17-19,24,25], two used erythromycin [16,21], one used clarithromycin [22], and two used roxithromycin [20,23]. Six out of ten trials used placebo as control [16-18,20,21,25]. The treatment duration ranged from 8 weeks to 24 months. Most patients included in these studies had a history of recurrent bronchiectasis exacerbations.

### Assessment of risk of bias

Among the included studies, 60% presented random sequence generation (6 of 10), 40% reported allocation concealment (4 of 10), 70% had blinded assessment of participants and personnel (7 of 10), 50% described blinding of outcome assessment (5 of 10), 80% reported incomplete outcome data (8 of 10), 90% described the selective reporting (9 of 10), and 80% were without other bias (8 of 10). Details of the quality assessment are presented in the supplemental material (see Additional file 4: Figure S1).

### Primary efficacy outcome

**Figure 2:**
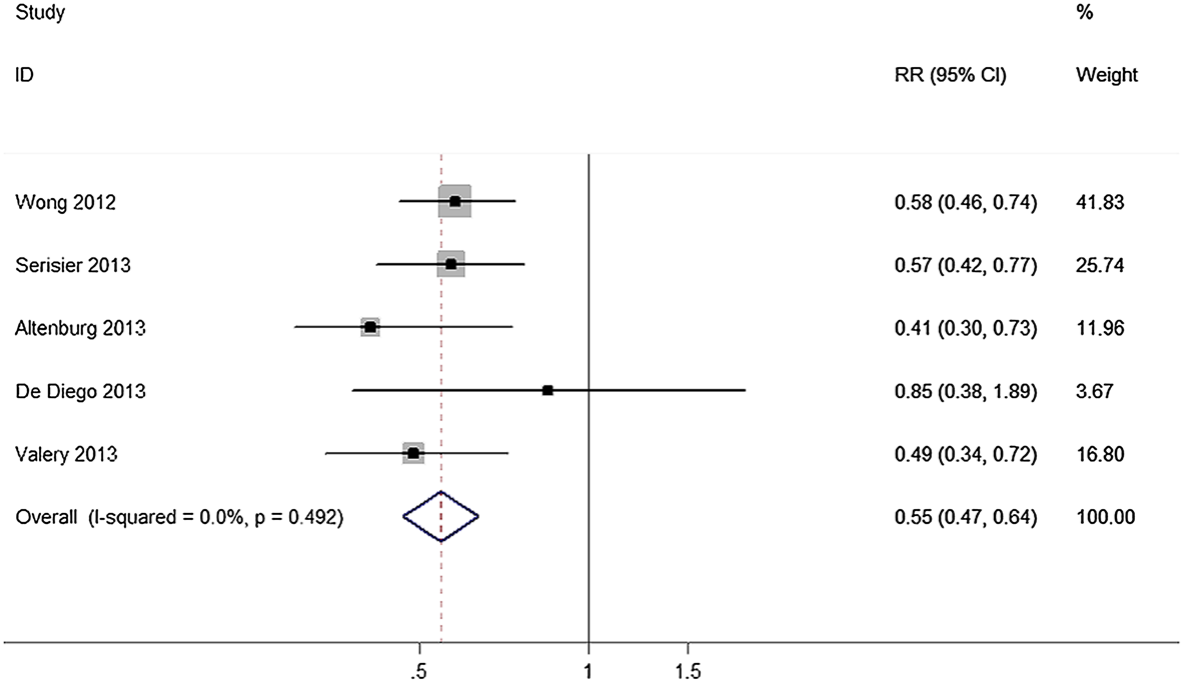
Analysis of the number of exacerbations. Forest plot assessed Odds Ratio (OR) of acute pulmonary exacerbations as a function of person-years in patients receiving macrolides compared to control.

Ten studies involving 601 non-CF bronchiectasis patients reported the primary efficacy outcome of macrolides on exacerbations. Macrolides treatment resulted in a significant reduction in the number of acute exacerbations per patient compared with control (RR = 0.55, 95% CI: 0.47 to 0.64, P value < 0.001, I^2^ = 0; Figure 2). When we restricted only to double blind studies and studies in adults, the pooled result was 0.55 (95%CI, 0.46-0.65, I^2^ = 0, p = 0.383) from three studies [16-18]. Table 1 presents the number of patients who had acute pulmonary exacerbations stratified by frequencies. Participants who were free from exacerbations significantly increased over placebo (OR = 2.81, 95% CI: 1.85 to 4.26, P value <0.001). No evidence of statistical heterogeneity was found for this outcome (I^2^ = 0, p = 0.43). In subgroup analysis, there was a statistically-significant decrease in the number of patients who had at least three exacerbations in the macrolides group (OR = 0.38, 95% CI: 0.22 to 0.65, P value = 0.0004, I^2^ = 0), but not in those who had one exacerbation (OR = 1.18, 95% CI: 0.70 to 2.01, P value = 0.53, I^2^ = 17%) or two exacerbations (OR = 0.83, 95% CI: 0.46 to 1.52, P value =0.55, I^2^ = 34%) compared with the control group. Relative forest plots could be found in Additional file 5: Figure S2. Overall, the pooled result of the number of participants who had at least one exacerbation was significantly decreased (OR = 0.36, 95% CI: 0.24 to 0.55, P value <0.01, I^2^ = 0%; see Additional file 6: Figure S3).

**Table 1:** **Analysis of number of patients stratifying by exacerbations**

**Exacerbations** ^**a**^	**No. of studies**	**Events/Total**		**Effect size**		**Heterogeneity**	
		**Macrolide**	**Control**	**OR(95%CI)**	**P value**	**I** ^**2**^ **(%)**	**P**
0	8	135/277	80/267	2.81(1.85-4.26)	<0.01 *	0	0.43
1	5	37/171	31/164	1.18(0.70-2.01)	0.53	17	0.31
2	3	24/147	27/142	0.83(0.46-1.52)	0.55	34	0.22
At least 3	3	34/147	60/142	0.38(0.22-0.65)	0.0004 *	0	0.45

^a^Number of exacerbations per patient; ^b^The subgroups of at least 1 exacerbations contributed to the overall estimate;* It was suggestive of statistical significance.

### Secondary efficacy outcomes

Time to a first exacerbation in the macrolide group was much greater than that in the control group (HR = 0.38, 95% CI: 0.28 to 0.53, P value < 0.001, I^2^ = 25.3%; see Additional file 7: Figure S4).

Four studies contributed to the meta-analysis of lung function. Changes of attenuation in FEV1 decline (weighted mean difference = 0.02, 95% CI, 0 to 0.04, P value = 0.02, I^2^ = 2%) and percent of predicted FEV1 (weighted mean difference = 1.52, 95% CI, 0.49 to 2.56, P value = 0.04, I^2^ = 0%) showed statistically-significant differences with the macrolides treatment compared with control. However, there was no significant difference in change of FVC between the two groups (weighted mean difference = 0.05, 95% CI, −0.03 to 0.13, P value = 0.25, I^2^ = 54%, Table 2).

**Table 2:** **Analysis of changes in lung function before and after treatment**

**Changes** ^**a**^	**No. of studies**	**No. of patients**		**Effect size**		**Heterogeneity**	
		**Macrolide**	**Control**	**WMD (95%CI)**	**P value**	**I** ^**2**^ **(%)**	**P value**
FEV1(L)	4	109	105	0.02(0–0.04)	0.02*	2	0.38
FEV1,% predicted	3	115	110	1.52(0.49-2.56)	0.004*	0	0.57
FVC(L)	3	98	95	0.05(−0.03-0.13)	0.25	54	0.11

Abbreviations: WMD, weighted mean difference; 95%CI, 95% confidence interval; FEV1, forced expiratory volume in the first second; FVC, forced vital capacity. ^a^change from baseline to the end of the study; * It was suggestive of statistical significance.

When patients were treated with macrolide antibiotics, improvement of quality of life was observed. Stratifying by the component of St George’s Respiratory Questionnaire (SGRQ), there were significant differences in the changes of SGRQ total score (weighted mean difference = −5.39, 95% CI, −9.88 to −0.89, P value = 0.02, I^2^ = 84%) and impact score (weighted mean difference = −5.88, 95% CI, −9.05 to −2.71, P value < 0.001, I^2^ = 36%). Although there was no significant difference in other subgroup analyses, there was a trend of improvement of quality of life in SGRQ symptoms (weighted mean difference = −13.38, 95% CI, −30.62 to 3.86, P value = 0.13) and activity score (weighted mean difference = −0.79, 95% CI, −4.67 to 3.09, P value = 0.69, Table 3). A significant difference in the change of dyspnea was also observed (weighted mean difference = −0.47, 95% CI, −0.57 to −0.37, P value < 0.001, I^2^ = 0, see Additional file 8: Figure S5).

**Table 3:** **Analysis of changes in quality of life during the study period**

**Changes** ^**a**^ **of SGRQ**	**No. of studies**	**No. of patients**		**Effect size**		**Heterogeneity**	
		**Macrolide**	**Control**	**WMD (95%CI)**	**P value**	**I** ^**2**^ **(%)**	**P value**
Total	5	213	204	−5.39(−9.88,-0.89)	0.02*	84	<0.001^b^
Symptoms	3	146	142	−13.38(−30.62,3.86)	0.13	94	<0.001^b^
Activity	2	87	84	−0.79(−4.67,3.09)	0.69	0	0.71
Impact	2	87	84	−5.88(−9.05,-2.71)	<0.001*	36	0.21

Abbreviations: WMD, weighted mean difference. ^a^changes from baseline to the end of the study. ^b^I^2^ > 50%, a random-effects model was chosen; *It was suggestive of statistical significance.

**Figure 3:**
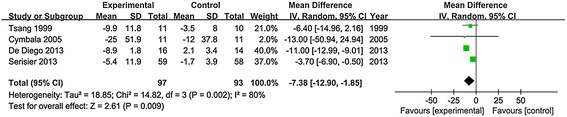
Analysis of the sputum volume. Forest plot assessed Odds Ratio (OR) of changes in 24-hour sputum volume before and after treatment between treatment group and control.

Macrolides maintenance treatments also resulted in decrease of sputum volume in non-CF bronchiectasis patients. Among four trials that included results for the change of 24-hour sputum volume, the weighted mean difference was −7.38 (95% CI, −12.90 to −1.85, P value = 0.009, I^2^ = 80%, Figure 3).

Macrolide treatments showed tremendous variations in the efficacy of pathogen eradication. In the six trials that assessed the eradication of *H. influenzae*, macrolide antibiotics maintenance therapy was associated with a significant benefit (OR = 2.06, 95% CI: 1.19 to 3.56, P value = 0.01, I^2^ = 27%, Table 4). In the three trials that assessed the eradication of *M. catarrhalis*, the result was also significant (OR = 2.95, 95% CI: 0.99 to 8.78, P value = 0.05, I^2^ = 38%). Analysis of any common pathogens from three studies resulted in an OR of 1.76 (95% CI, 0.91 to 3.41,P = 0.09). No significant heterogeneity was detected (P = 0.23).

**Table 4:** **Analysis of distribution of pathogens during study period**

**Pathogens**	**No. of studies**	**Events/Total**		**Effect size**		**Heterogeneity**	
		**Macrolide**	**Control**	**OR(95%CI)**	**P**	**I** ^**2**^ **(%)**	**P**
**Eradication** ^**a**^							
Any^b^	3	39/104	28/99	1.76(0.91,3.41)	0.09	31	0.23
H. i	6	44/219	23/208	2.06(1.19,3.56)	0.01*	27	0.23
P. a	4	20/161	15/154	1.32(0.63,2.77)	0.46	0	0.63
S. a	3	5/106	7/102	0.69(0.22,2.12)	0.51	0	0.45
S. p	2	4/63	7/62	0.50(0.13,1.94)	0.32	38	0.20
M. c	3	13/130	4/122	2.95(0.99,8.87)	0.05*	38	0.20
**Newly appearance**							
Any^b^	4	28/163	36/157	0.68(0.37,1.23)	0.20	0	0.51
H. i	4	14/163	17/157	0.79(0.38,1.63)	0.52	61	0.05
P. a	2	6/105	6/103	0.98(0.32,3.02)	0.97	35	0.21
S. a	2	3/87	1/82	1.9(0.34,10.61)	0.47	47	0.17
S. ${\mathrm{p}}_{=}^{\mathrm{d}}$	1	2/46	0/45	5.11(0.24,109.51)	0.30	-	-
M. c	2	0/87	6/82	0.12(0.01,1.04)	0.05*	0	0.97

Abbreviations: H. i, Haemophilus influenzae; P. a, Pseudomonas aerugiosa; S. a, Staphylococcus aureus; S. p, Streptococcus pneumoniae; M. c, Moraxella catarrhalis; OR (95%CI), Odds Ratio (95% confidential interval). ^a^Pathogens reported eradicated during study period; ^b^any of the pathogens reported in the included studies; ^d^There was only one study reported S.p resistance, so Heterogeneity of this subgroup could not analysis; *It was suggestive of statistical significance.

In a meta-analysis of the new appearance of five common pathogens, any reported pathogens showed no statistically-significant difference between the two groups (OR = 0.68, 95% CI: 0.37 to 1.23, P value = 0.20, I^2^ = 0%). However, there was a significant risk reduction of new emergence of *M. catarrhalis* in patients treated with macrolides (OR = 0.12, 95% CI: 0.01 to 1.04, P value = 0.05, I^2^ = 0, Table 4).

In terms of the microbiologic profile detected in the respiratory secretions at the end of the study, there was a highly statistically-significant reduction in *M. catarrhalis* (OR = 0.15, 95% CI: 0.04 to 0.60, P value = 0.007, I^2^ = 0) and *S. pneumoniae* (OR = 0.41, 95% CI: 0.18 to 0.91, P value = 0.03, I^2^ = 52) among patients taking long-term macrolides versus the control group. The reported organisms showed that there was no statistically-significant difference (OR = 0.73, 95% CI: 0.41 to .131, P value = 0.30, I^2^ = 0%; see Additional file 3: Table S2).

### Safety outcomes

**Figure 4:**
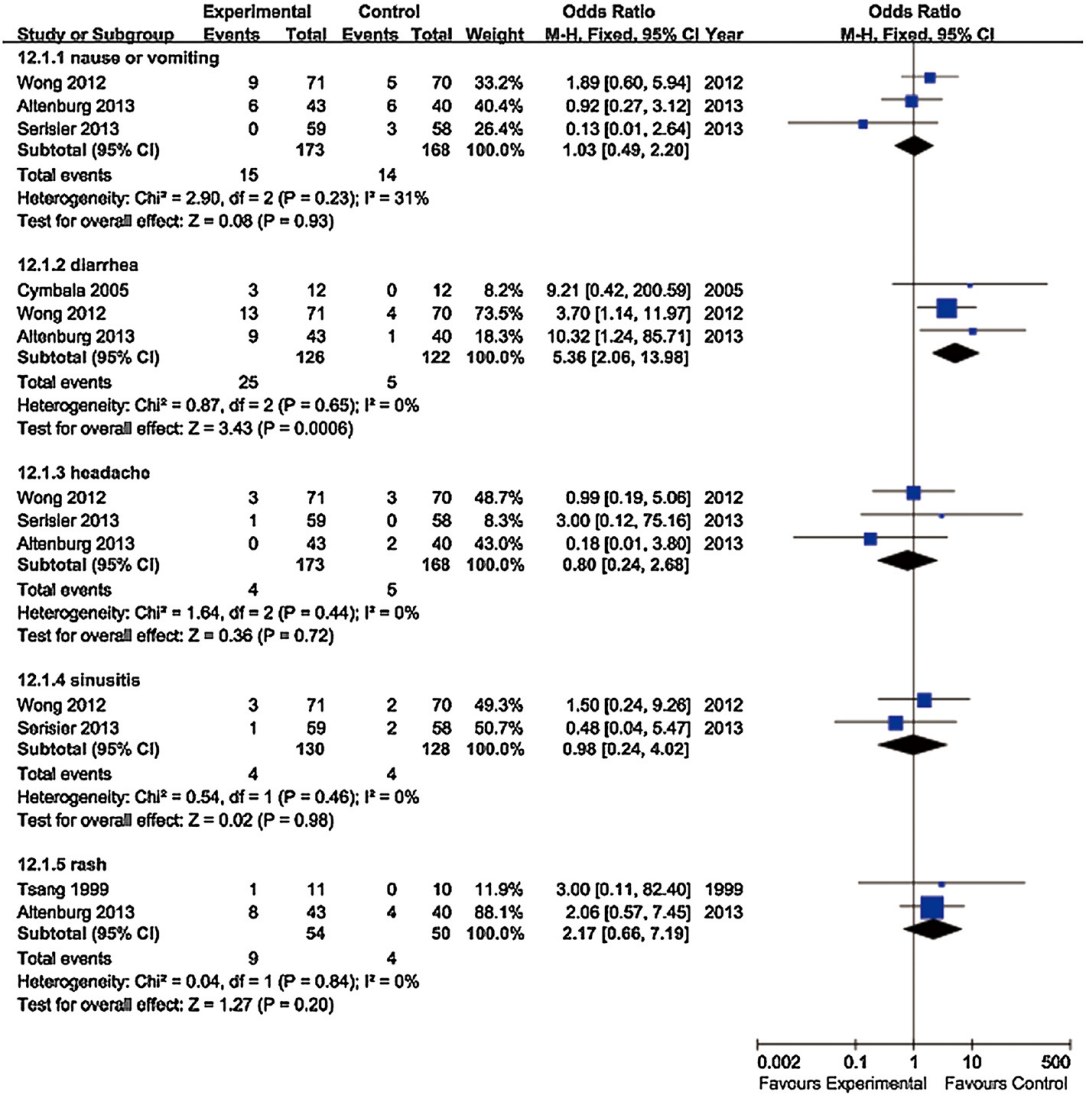
Analysis of adverse effects. Forest plot assessed Odds Ratio (OR) of the main adverse effects among non-CF bronchiectasis patients receiving macrolides compared to control.

Side effects reported by five articles were assessed. The main adverse effects were analyzed in subgroups (Figure 4). The risk of diarrhea was found to be statistically higher among participants receiving macrolides compared to those receiving placebo (OR = 5.36, 95% CI: 2.06 to 13.98, P value = 0.0006, I^2^ = 0%). Incidences of nausea or vomiting (OR = 1.03, 95% CI: 0.49 to 2.20, P value = 0.93, I^2^ = 31%), headache (OR = 0.80, 95% CI: 0.24 to 2.68, P value = 0.72, I^2^ = 0%), sinusitis (OR = 0.98, 95% CI: 0.24 to 4.02, P value = 0.98, I^2^ = 0%) and rash (OR = 2.17, 95% CI: 0.66 to 7.19, P value = 0.20, I^2^ = 0%) were not statistically different between the macrolides group and placebo group.

In the four trials that reported patient withdrawal because of adverse events, no statistical difference was found in terms of participant withdrawal due to adverse events among patients taking prophylactic macrolides compared to those taking placebo (OR = 1.18, 95% CI: 0.33 to 4.19, P value = 0.80, I^2^ = 0%; see Additional file 9: Figure S6).

**Figure 5:**
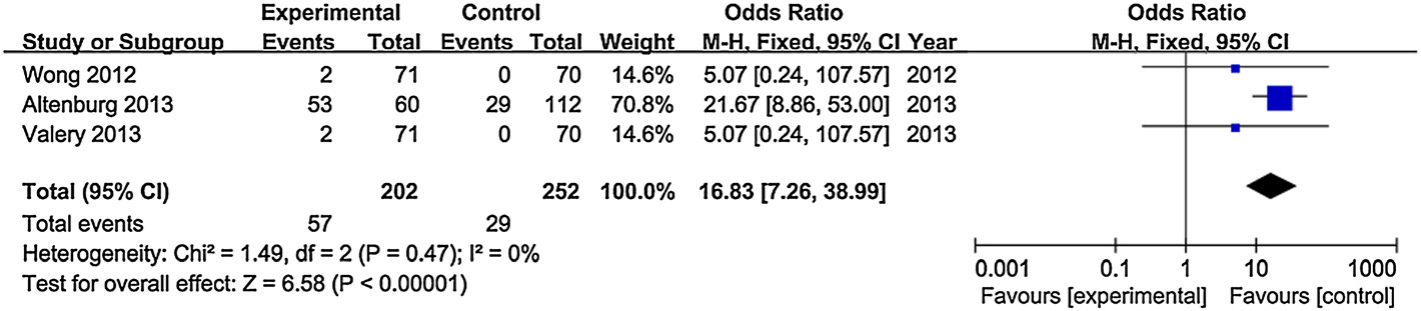
Analysis of macrolide resistance. Forest plot assessing odds ratio (OR) of any antimicrobial resistance caused by macrolide among non-CF bronchiectasis patients receiving macrolides compared to control.

Three studies reported pathogen resistance caused by the usage of macrolide antibiotics. Significant difference was observed with macrolides treatment, compared with control (OR = 16.83, 95% CI: 7.26 to 38.99, P value < 0.001, I^2^ = 0%, Figure 5). When patients were treated with macrolides, there was an increased risk of macrolide resistance in *H. influenzae* (OR = 67.47, 95% CI: 8.49 to 536.02, P value < 0.001), *S. aureus* (OR = 5.91, 95% CI: 2.01 to 17.35, P value = 0.001, I^2^ = 0%) and *S. pneumoniae* (OR = 11.74, 95% CI: 2.46 to 56.03, P value = 0.002, I^2^ = 0%). The pooled estimate involved five pathogens and was significantly different (OR = 6.45, 95% CI: 1.81 to 23, P value = 0.004, I^2^ = 64%, see Additional file 10: Figure S7).

## Discussion

Patients with non-CF bronchiectasis suffer from recurrent exacerbations, resulting in the destruction of the airways and reduced quality of life [26]. This meta-analysis presented evidence for a beneficial effect on pulmonary exacerbation with macrolides treatment. For the number of acute exacerbations per patient, our results show a statistically-significant reduction. Although the meta-analysis performed by Gao was also assessed the efficacy and safety of macrolides in patients with non-cystic fibrosis bronchiectasis [27], we actually conducted from different perspectives. For the primary outcome, although both of us concluded that macrolide therapy could significantly decrease the number of patients with exacerbations. In Gao’s study, it assessed the number of patients with experiencing at least one exacerbations, while we analyzed the number of patients stratifying by different exacerbations (Table 1). And we found that the number of participants free from exacerbation was significantly greater, and the number of participants who had at least 3 exacerbations was significantly less in macrolide-treated group compared with control group. Meanwhile, there was no statistical significance of the number of participants had one and two exacerbations between the two groups. The details of these results were not reflected by Gao’s study. There was an increased trend of patients experiencing just one exacerbation in the macrolide group compared with control, which is attributed to the fact that more patients were “restricted” to one exacerbation. More patients were having only 1 but not 2 or 3 exacerbations due to the usage of macrolides. A meta-analysis performed by Shi et al. showed that the number of patients who had at least one exacerbation was significantly greater in the macrolide treated group [28]. However, the incorrect extraction of data affected the overall accuracy and validity of this meta-analysis, which was also pointed out by Serisier and Gao [29,30].

In addition, time to a first exacerbation was significantly prolonged in patients taking macrolides compared with placebo. Results for the outcomes suggest that macrolides can affect the frequency of exacerbations in patients with non-CF bronchiectasis. Of note, in addition to the eight RCTs which suggested a significant reduction in non-CF bronchiectasis exacerbations among patients taking long term macrolides, three additional studies showed similar findings [31-33]. Reduction of exacerbations in patients with non-CF bronchiectasis contributed to the attenuation of lung function decline and improvement in quality of life. As well, both of us found the quality of life was improved in macrolide group. We evaluated the effects of macrolide therapy on quality of life stratified by all components of St George’s Respiratory Questionnaire (SGRQ). And we found that there were significant differences in the changes of SGRQ total score (weighted mean difference = −5.39, 95% CI, −9.88 to −0.89, P value = 0.02, I^2^ = 84%) and impact score (weighted mean difference = −6.13, 95% CI, −8.52 to −3.74, P value < 0.001, I^2^ = 36%). But there was no significant difference in SGRQ symptoms score (weighted mean difference = −13.38, 95% CI, −30.62 to 3.86, P value = 0.13) and activity score (weighted mean difference = −0.79, 95% CI, −4.67 to 3.09, P value = 0.69, Table 3). While only SGRQ total score was assessed in Gao’s study. Additionally, we found the 24-hour sputum volume was significantly decreased in patients taking macrolides compared with placebo (weighted mean difference = −7.38, 95% CI, −12.90 to −1.85, P value = 0.009, Figure 3). The reduction of sputum volume also benefits the improvement of lung function. The reduction of sputum production could be associated with the inhibitory effect of respiratory glycoconjugate exerted by macrolides [34].

These clinical benefits of macrolides in the treatment of non-CF bronchiectasis may be associated with a lower bacterial load. In an analysis of bacterial characteristics before and after treatment, the pooled estimate showed that there was a significantly increased eradication of pathogens in the macrolide group (p < 0.05), while there was no significant difference in the new emergence of pathogens between the two groups. Meta-analysis also showed a statistically-significant reduction of pathogens in patients treated with macrolide antibiotics at the end of the study. A meta-analysis of long-term azithromycin use in patients with chronic lung diseases showed that azithromycin might decrease colonization of bacteria (RR = 0.551, 95%CI, 0.46, 0.658, P < 0.001), which is consistent with our findings [35]. The lower bacterial load during long-term macrolide treatment is considered to be associated with its immunomodulatory and antibiotic activities. Macrolides exert their activity by inhibiting neutrophil recruitment, chemical mediator release, virulence factors production and quorum sensing functions [9].

An important matter of concern in the widespread implementation of the long-term use of macrolides is their adverse effects. In this meta-analysis, we found that the pooled estimate of side effects was significantly greater in the macrolides treatment group than that in the control group (P = 0.01). Significantly increased risk of diarrhea was observed among patients treated with macrolides. However, it was mostly mild. The incidence of nausea or vomiting, headache, sinusitis and rash showed no statistically-significant differences between the macrolides treatment group and the control group. In addition, no statistically-significant difference between the two groups was found in the number of patients discontinuing the study due to an adverse event. Ray et al. reported a small absolute increase in cardiovascular deaths during 5 days of azithromycin therapy [36]. Trials addressing this issue were rare; only the BLESS study reported that there was no evidence of macrolides causing QTc prolongation or arrhythmia. However, to prevent cardiovascular events, care should be taken and recording ECG to monitor the QT interval should be recommended in clinical practice.

Another critical issue limiting the use of long-term macrolides therapy is the risk of induction of resistant bacterial strains, which was especially pointed out by Serisier DJ [37,38]. Compared with study performed by Wu *et al.*, our study attempt to strengthen evidence on the macrolide resistance risk [39]. To our knowledge, none of the meta-analysis evaluating this important issue until now. Although trials reported this issue were rare, we tried to gain some evidence to this important issue. By meta-analysis, our results showed that macrolides use was associated with a statistically-significant increase of antimicrobial resistance. An increased risk of development of macrolide-resistant *S. pneumonia*, *S. aureus* and *H. influenczae* was observed in the meta-analysis. In subgroup analyses, there was no statistically-significant difference of eradication in *S. pneumonia* and *S. aureus*, which may be associated with drug resistance, deficiency of dosage or limitation on the bioactivities of macrolides.

The world-wide prevalence of macrolide resistance in *S. pneumoniae* was 16.5% in 1996, 21.9% in 1997 [40] and 24.6% during 1998–2000 [41]. The rapidly increasing rate of macrolide resistance in *S. pneumonia* was coincident with increased macrolide sales [37]. Carriage of the resistant strain is not only a risk to individuals but also a threat to the community. A study suggested that penicillin resistance was associated three times more strongly with macrolides than with penicillins [42]. There was evidence that suggested that the increase in penicillin resistance associated with macrolide use was due to the carriage of co-resistant strains [42,43]. Transfer of the resistance gene between strains may be responsible for the emergence of multidrug resistance among different bacteria [44]. Emergence of multi-drug resistant strains is a great risk to the whole community. Therefore, in clinical practice, the physician should be more cautious to select the appropriate patients and weigh the clinical benefit against the risks.

### Study limitations

There were some limitations of our meta-analysis. First, the overall number of patients included in our review was relatively small. Although we tried to collect all the relevant data, it is hard to ensure that no data were missed. Secondly, the enrolled patients of each study had different exacerbations in the past year before inclusion and were in different stages of disease. Nevertheless, it should be noted that all the included studies were RCTs and the most pooled results showed no statistically-significant heterogeneity, which could partly make up for the defect. Thirdly, treatments of macrolides used in the included studies were of different types, dosages and durations. The paucity of studies made it difficult to determine the dose–response relationship between macrolides and benefits.

## Conclusions

Macrolide maintenance treatment could reduce acute pulmonary exacerbations, decrease sputum production, attenuate lung function decline, improve quality of life and increase the eradication of pathogens. Meanwhile, macrolide maintenance treatment was associated with an increase in the risk of side effects and antimicrobial resistance. Patients with frequent exacerbations are prone to be considered to be prescribed long-term macrolide therapy. They should be carefully evaluated during the follow up treatment. A balance between clinical benefit and potential development of macrolides resistance in pathogens and adverse events should be well weighed. Novel synthetically-derived macrolides that preserve anti-inflammatory functions as well as overcome the risk of microbial resistance are needed to be investigated for the long-term treatment of chronic inflammatory respiratory diseases. More randomized controlled trials involving larger patient samples are warranted to confirm the appropriate dosage and duration of macrolides for non-CF bronchiectasis patients.

## Additional files

Additional file 1:
**PROSPERO 2013 (CRD42013004656).**
Additional file 2:
**PRISMA checklist.** Information on how this meta-analysis was conducted. Additional file 3: Table S1. Characteristics of studies included in the meta-analysis. **Table S2.** Analysis of microbial distribution at the end of the study. Additional file 4: Figure S1. Quality assessment of included studies. The quality assessment of each study was according to the Cochrane Collaboration tool in the Review Manager software. Additional file 5: Figure S2. Forest plot of the number of patients who had acute pulmonary exacerbations stratified by frequencies. Additional file 6: Figure S3. Forest plot of the number of participants who had at least one exacerbation. Additional file 7: Figure S4. Analysis of time to a first exacerbation. Forest plot assessing hazard ratio (HR) of time to a first exacerbation among patients receiving macrolides compared to placebo. Additional file 8: Figure S5. Forest plot assessing changes of dyspnea. Additional file 9: Figure S6. Analysis of participants withdrawal owing to side effects. Forest plot assessing odds ratio (OR) of withdrawal from study due to an adverse event among patients receiving macrolides compared to placebo. Additional file 10: Figure S7. Analysis of antimicrobial resistance stratifying by pathogens. Forest plot assessing odds ratio (OR) of five different antimicrobial resistance induced by macrolide antibiotics in the treatment group and the control.

## Abbreviations

non-CF: Non-cystic fibrosis bronchiectasis
OR: Odds ratios
95%CI: 95% Confidence interval
FEV1: Forced expiratory volume in the first second
FVC: Forced vital capacity
BALF: Bronchoalveolar lavage fluid
SGRQ: St George’s Respiratory Questionnaire
RCT: Randomized controlled trial
