# Supplementary material for: Effects of long-term use of macrolides in patients with non-cystic fibrosis bronchiectasis: a meta-analysis of randomized controlled trials
Source: BMC Infect Dis. 2015 Mar 27;15:160. doi: 10.1186/s12879-015-0872-5 (PMC4464873; doi:10.1186/s12879-015-0872-5)
Supplement: Additional file 1: — PROSPERO 2013 (CRD42013004656). [file 12879_2015_872_MOESM1_ESM.pdf]

Efficacy and safety of prolonged use of macrolide in patients with noncystic fibrosis bronchiectasis: a meta-analysis

Li-Chao Fan, Jin-Fu Xu, Hui-Ping Li, Hai-Wen Lu, Xiao-Bin Ji

Citation

Li-Chao Fan, Jin-Fu Xu, Hui-Ping Li, Hai-Wen Lu, Xiao-Bin Ji. Efficacy and safety of prolonged use of macrolide in patients with noncystic fibrosis bronchiectasis: a meta-analysis. PROSPERO 2013:CRD42013004656 Available from http://www.crd.york.ac.uk/PROSPERO_REBRANDING/display_record.asp?ID=CRD42013004656

Review question(s)

Is the prolonged use of macrolide antibiotic in patients with noncystic fibrosis bronchiectasis safe and efficient?

Searches

PubMed, EMBASE, Web of Science and the Cochrane Library were searched by two investigaters. Search terms included “Macrolide antibiotics" or “azithromycin” or “erythromycin” or “clarithromycin” or “roxithromycin” and "bronchiectasis". In addition, a manual search of relevant articles was also performed.

Types of study to be included

Inclusion criteria: RCTs, published or unpublished.

Exclusion criteria: open-label.

Condition or domain being studied

Bronchiectasis is a chronic lung disease postulated to be chronic bronchial sepsis and persistent airway inflammation.The pathophysiology of noncystic fibrosis bronchiectasis (suptum, PA. ,imflammasome, destruction of airway, vicious cycle). Standard therapies for noncystic fibrosis bronchiectasis include antibiotics, especially macrolides. Due to their anti-inflammatory and anti-bacterial effects, macrolides show great potential as an adjunct treatment for noncystic fibrosis bronchiectasis. However, the efficacy and safety of macrolide therapy in noncystic fibrosis bronchiectasis patients are the subject of controversy.

Participants/ population

Inclusion criteria: people included in the analysis fulfilled strict criteria for the diagnosis of noncystic fibrosis bronchiectasis.

Exclusion criteria: patients with chronic respiratory conditions other than noncystic fibrosis bronchiectasis, such as cystic fibrosis.

Intervention(s), exposure(s)

Inclusion criteria: use of a prolonged macrolide antibiotic(where prolonged is two months or longer) compared to controls who receive placebo.

Exclusion criteria: the days of macrolide antibiotic therapy is less than two months.

Comparator(s)/ control

Inclusion criteria: macrolide antibiotic compared to controls who receive placebo.

Exclusion criteria: studies that have no controls using placebo.

Outcome(s)

Primary outcomes

Changes from baseline of pulmonary function tests and number of exacerbations.

Pulmonary function tests: the forced expiratory volume in 1 second (FEV1) (L) or FEV 1% of predicted value (%).

Secondary outcomes

Changes from baseline of SGRQ score, 6-min walk test distance(m), adverse effects, macrolide resistant, CRP.

Risk of bias (quality) assessment

The risk of bias for each identified trial will be assessed in accordance with the Cochrane Collaboration tool for the assessment of these variables. Six components will be assessed:

(1) adequate sequence generation;

(2) allocation concealment;

(3) blinding;

(4) incomplete outcome data addressed;

(5) free of selective reporting; and

(6) free of other bias.

Strategy for data synthesis

Weighted mean difference will be used for pulmonary function tests, and risk ratios for all other variables (SGRQ score, 6-min walk test distance(m), adverse effects, macrolide resistant, CRP)

Analysis of subgroups or subsets

Duration of therapy less than or more than six months.

Contact details for further information

Li-Chao Fan

Shanghai Pulmonary Hospital

Tongji University School of Medicine

Shanghai 200433

China

fanlichao2012@126.com

Organisational affiliation of the review

none

Review team

Dr Li-Chao Fan, Shanghai Pulmonary Hospital
Dr Jin-Fu Xu, Shanghai Pulmonary Hospital
Dr Hui-Ping Li, Shanghai Pulmonary Hospital
Dr Hai-Wen Lu, Shanghai Pulmonary Hospital
Dr Xiao-Bin Ji, Shanghai Pulmonary Hospital

Anticipated or actual start date

03 May 2013

Anticipated completion date
30 August 2013

Funding sources/sponsors
the National Science Foundation of China [NSFC81170003]; Shanghai Pujiang Project [12PJD004] and Shanghai Elite Medical Talent Project [XYQ2011006].

Conflicts of interest
None known

Language
English

Country
China

Subject index terms status
Subject indexing assigned by CRD

Subject index terms
Bronchiectasis; Humans; Lung Diseases; Macrolides; Respiratory Tract Infections

Date of registration in PROSPERO
21 May 2013

Date of publication of this revision
21 May 2013

**Stage of review at time of this submission**	**Started**	**Completed**
Preliminary searches	Yes	No
Piloting of the study selection process	Yes	No
Formal screening of search results against eligibility criteria	No	No
Data extraction	No	No
Risk of bias (quality) assessment	No	No
Data analysis	No	No

PROSPERO
International prospective register of systematic reviews
The information in this record has been provided by the named contact for this review. CRD has accepted this information in good faith and registered the review in PROSPERO. CRD bears no responsibility or liability for the content of this registration record, any associated files or external websites.
