# Supplementary material for: Effects of long-term use of macrolides in patients with non-cystic fibrosis bronchiectasis: a meta-analysis of randomized controlled trials
Source: BMC Infect Dis. 2015 Mar 27;15:160. doi: 10.1186/s12879-015-0872-5 (PMC4464873; doi:10.1186/s12879-015-0872-5)
Supplement: Additional file 3: Table S1. — Characteristics of studies included in the meta-analysis. Table S2. Analysis of microbial distribution at the end of the study. [file 12879_2015_872_MOESM3_ESM.doc]

**Supplementary tables**

**Table S1 Characteristics of studies included in the meta-analysis.**

|  |  |  | **No. of patients (M/F)** | | **Mean age, y(SD)^a^** | |  |  |
| --- | --- | --- | --- | --- | --- | --- | --- | --- |
| **Source** | **Location** | **Design** | **treatment** | **control** | **treatment** | **control** | **intervention** | **duration** |
| Koh 1997[20] | Korea | RDBPCT | 7/6 | 7/5 | 13.3(2.5) | 12.9(2.6) | ROM 4mg/kg b.i.d. or placebo | 12 weeks |
| Tsang 1999[21] | Hong Kong | RDBPCT | 3/8 | 2/8 | 50(15) | 59(16) | ERM 500 mg b.i.d or placebo | 8 weeks |
| Cymbala 2005 | USA | ROC | 5/6 | 5/6 | 70.8(9.7) | 70.8(9.7) | AZM 500mg twice weekly | 6 months |
| Yalcın 2006[22] | Turkey | RCT | 8/9 | 11/6 | 13.1(2.7) | 11.9(2.9) | CLM 15 mg/kg daily | 3 months |
| Wong 2012[17] | New Zealand | RDBPCT | 23/48 | 20/50 | 60.9(13.6) | 59.0(13.3) | AZM 500 mg 3 times weekly or placebo | 6 months |
| Liu 2012 | China | RCT | 12/13 | 14/11 | 47(8) | 49(9) | ROM 150mg daily | 6 months |
| Altenburg 2013[18] | Netherlands | RDBPCT | 18/25 | 12/28 | 59.9(12.3) | 64.6(9.1) | AZM 250mg daily or placebo | 12months |
| Serisier 2013[16] | Australian | RDBPCT | 21/38 | 25/33 | 50(15) | 59(16) | ERM 250mg bid or placebo | 12months |
| De Diego 2013[19] | Spain | RCT | 7/9 | 7/7 | 57(11) | 61 (12) | AZM 250mg 3 times weekly | 3 months |
| Valery 2013 | New Zealand Australian | RDBCT | 26/19 | 21/23 | 3.99(2.14) | 4.22(2.30) | AZM 30mg/kg once a week | 24months |

Abbreviations: bid, twice daily; M/F, Male/Female; RDBPCT, randomised double-blind placebo-controlled trial; HRCT, high-resolution computed tomography; ERM, Erythromycin; AZM, Azithromycin; CLM, clarithromycin; ROM, roxithromycin; RCT, randomized controlled trial; ROC: randomized open-label crossover study.
^a^Data are mean years (SD).

**Table S2 Analysis of microbial** distribution at the end of the study.

| **Pathogens** | **No. of studies** | **Events/Total** | | **Effect size** | | **Heterogeneity** | |
| --- | --- | --- | --- | --- | --- | --- | --- |
|  |  | **Macrolide** | **Control** | **OR(95%CI)** | **P** | **I^2^ (%)** | **P** |
| Any^a^ | 3 | 58/101 | 61/94 | 0.73(0.41,1.31) | 0.30 | 0 | 0.83 |
| H. i | 6 | 35/204 | 46/200 | 0.70(0.43,1.14) | 0.15 | 61 | 0.03 |
| P. a | 5 | 37/173 | 30/167 | 1.28(0.71,2.32) | 0.41 | 0 | 0.64 |
| E. c | 2 | 1/54 | 1/50 | 0.92(0.12,6.80) | 0.94 | 0 | 0.32 |
| S. p | 4 | 9/137 | 21/135 | 0.41(0.18,0.91) | 0.03* | 52 | 0.10 |
| M. c | 4 | 1/137 | 13/135 | 0.15(0.04,0.60) | 0.007 | 0 | 0.84 |

Abbreviations: H. i, Haemophilus influenzae; P. a, Pseudomonas aerugiosa; E. c, Escherichia coli; S. p, Streptococcus pneumoniae; M. c, Moraxella catarrhalis; Odds Ratio (95% confidential interval). ^a^any of the pathogens reported in the included studies;
