# Supplementary figures and images for: Effects of long-term use of macrolides in patients with non-cystic fibrosis bronchiectasis: a meta-analysis of randomized controlled trials
Source: BMC Infect Dis. 2015 Mar 27;15:160. doi: 10.1186/s12879-015-0872-5 (PMC4464873; doi:10.1186/s12879-015-0872-5)

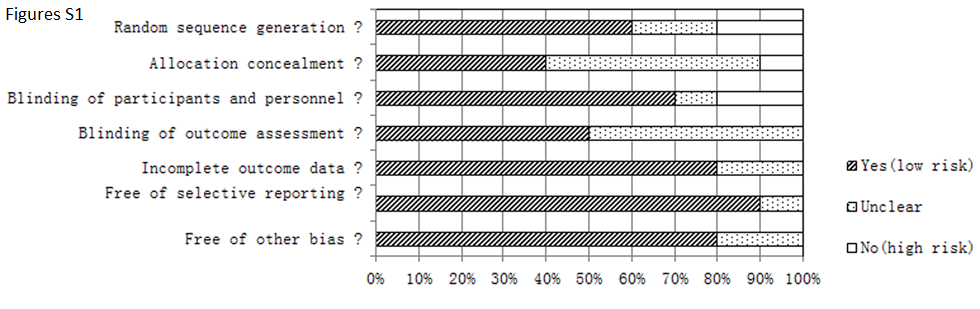

Supplement: Additional file 4: Figure S1. — Quality assessment of included studies. The quality assessment of each study was according to the Cochrane Collaboration tool in the Review Manager software. [file 12879_2015_872_MOESM4_ESM.tiff]

Figure S2

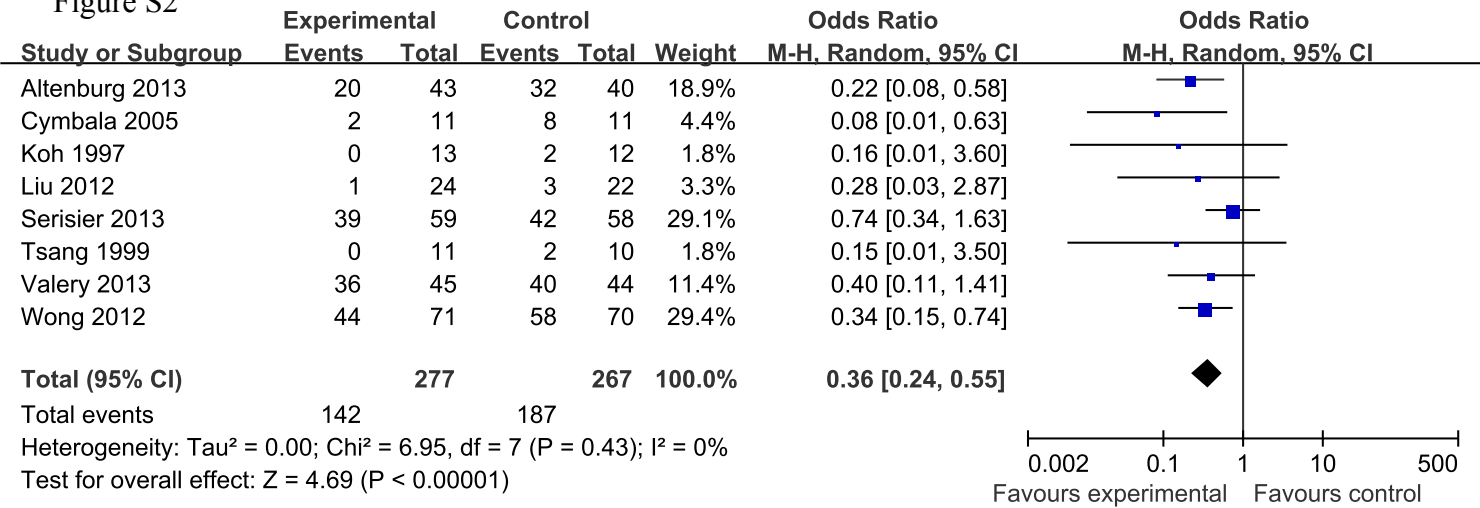

Supplement: Additional file 5: Figure S2. — Forest plot of the number of patients who had acute pulmonary exacerbations stratified by frequencies. [file 12879_2015_872_MOESM5_ESM.pdf]

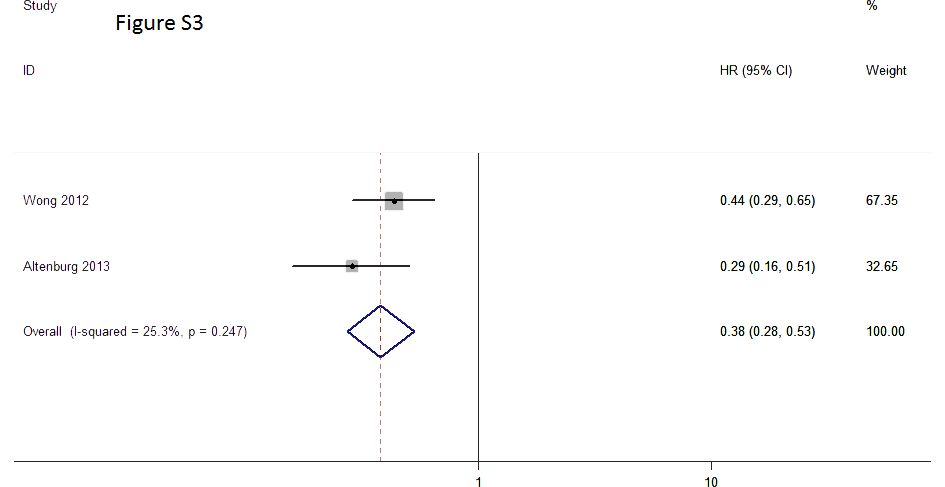

Supplement: Additional file 6: Figure S3. — Forest plot of the number of participants who had at least one exacerbation. [file 12879_2015_872_MOESM6_ESM.tiff]

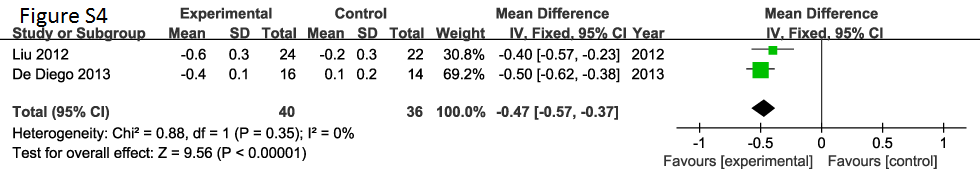

Supplement: Additional file 7: Figure S4. — Analysis of time to a first exacerbation. Forest plot assessing hazard ratio (HR) of time to a first exacerbation among patients receiving macrolides compared to placebo. [file 12879_2015_872_MOESM7_ESM.tiff]

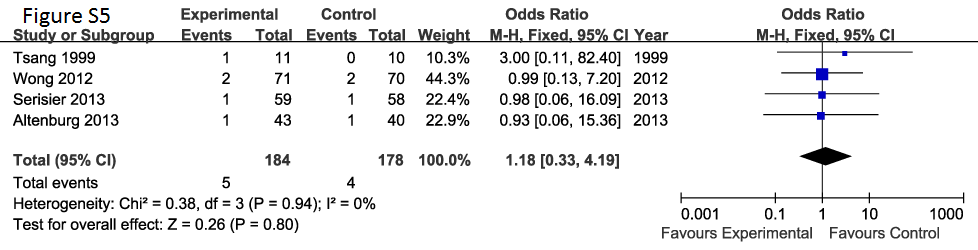

Supplement: Additional file 8: Figure S5. — Forest plot assessing changes of dyspnea. [file 12879_2015_872_MOESM8_ESM.tiff]

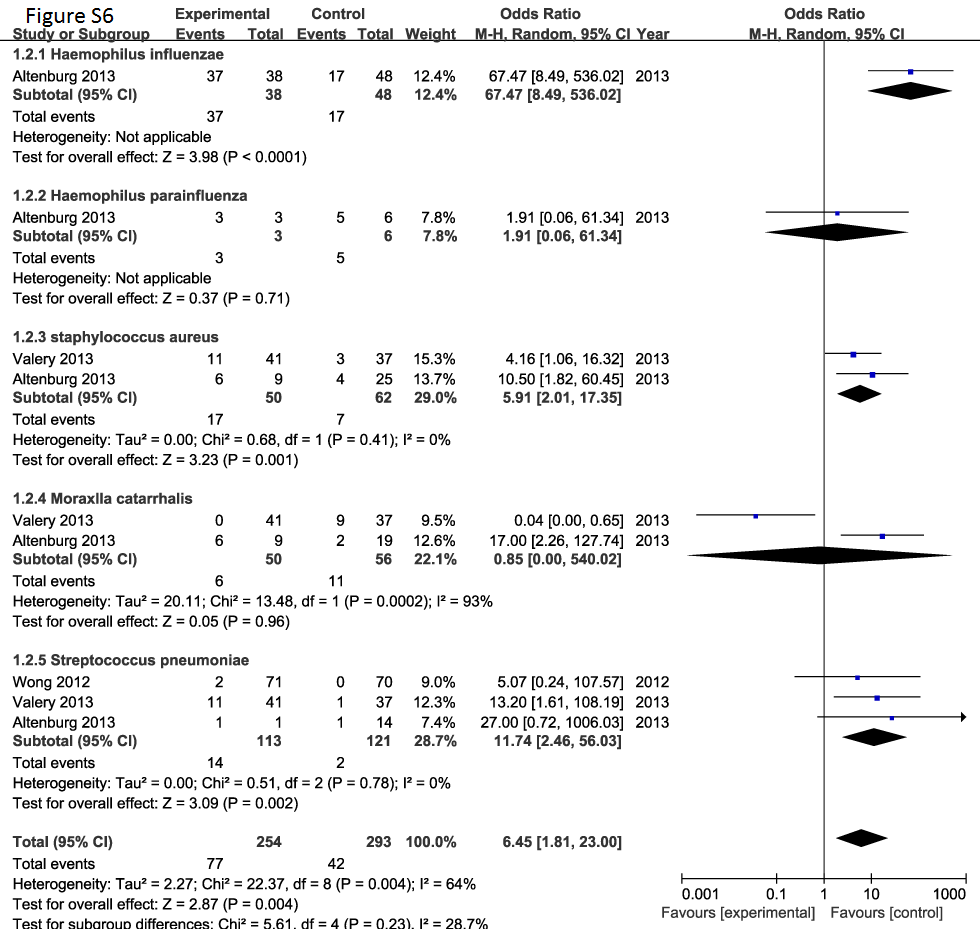

Supplement: Additional file 9: Figure S6. — Analysis of participants withdrawal owing to side effects. Forest plot assessing odds ratio (OR) of withdrawal from study due to an adverse event among patients receiving macrolides compared to placebo. [file 12879_2015_872_MOESM9_ESM.tiff]

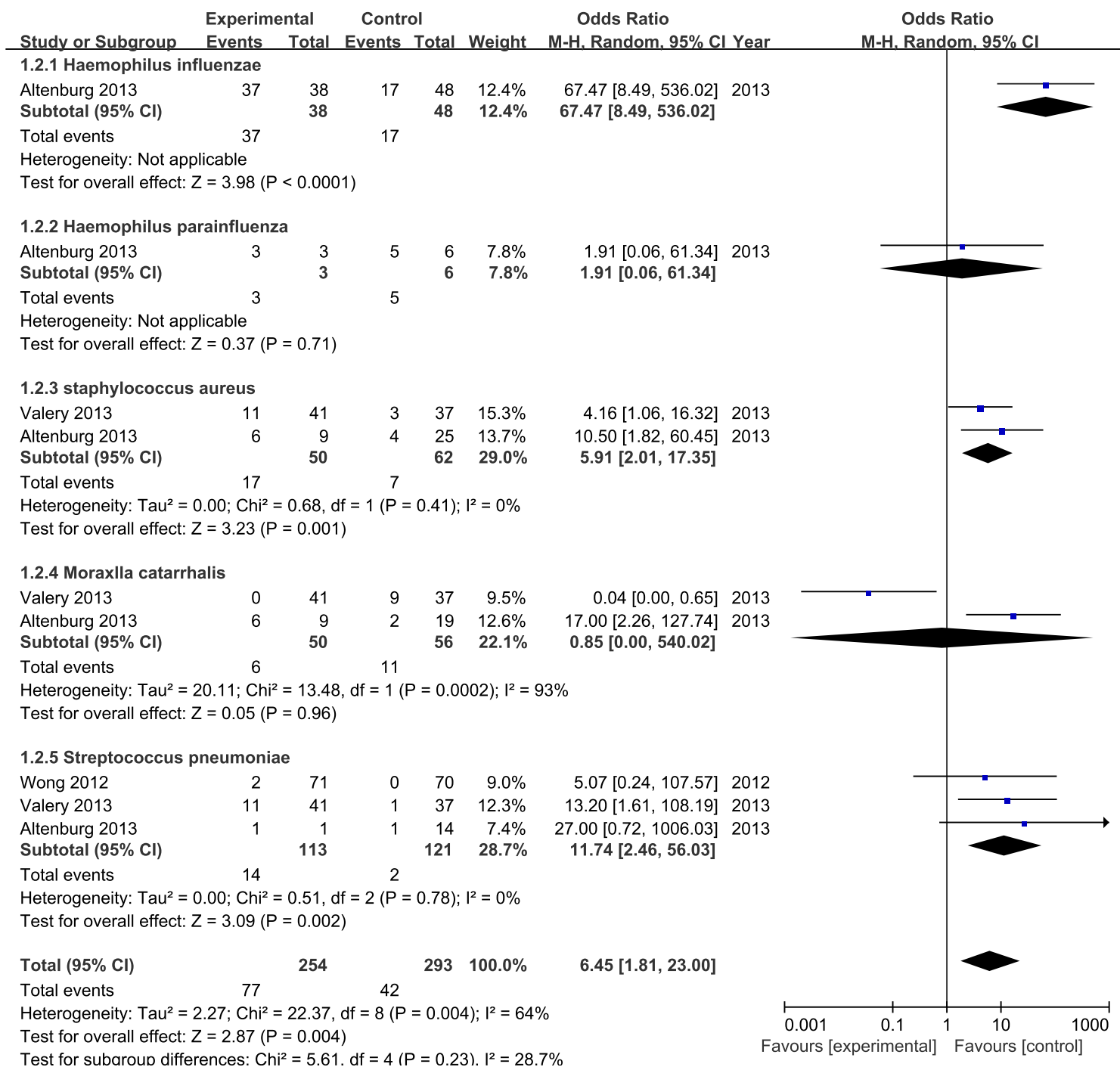

Supplement: Additional file 10: Figure S7. — Analysis of antimicrobial resistance stratifying by pathogens. Forest plot assessing odds ratio (OR) of five different antimicrobial resistance induced by macrolide antibiotics in the treatment group and the control. [file 12879_2015_872_MOESM10_ESM.pdf]
